# Case report: A case of primary angiitis of the central nervous system: misdiagnosed for 3.5 years

**DOI:** 10.3389/fneur.2023.1337410

**Published:** 2023-12-14

**Authors:** Lixia Qin, Miao He, Wei Lu

**Affiliations:** Department of Neurology, The Second Xiangya Hospital, Central South University, Changsha, Hunan, China

**Keywords:** PACNS, serial MRIs, tumor-like presentation, brain biopsy, mitochondrial encephalomyopathy, misdiagnosis

## Abstract

**Introduction:**

Primary angiitis of the central nervous system (PACNS) is an uncommon inflammatory condition that exclusively affects blood vessels within the brain parenchyma, leptomeninges, and spinal cord. Due to its infrequency and the variability in its clinical presentation and imaging findings, diagnosing PACNS can be challenging.

**Case description:**

In this study, we present the case of a teenager who initially presented with headaches and epilepsy. Comprehensive laboratory tests yield normal results. A series of brain magnetic resonance imaging (MRI) revealed a progression of changes, starting from localized cerebral atrophy and culminating in the development of a contrast-enhanced mass with vasogenic edema. Immune-associated encephalitis and mitochondrial encephalopathy were suspected, but immunologic investigations, mitochondrial DNA (mtDNA) and nuclear DNA (nDNA) sequencing using biopsied muscle, and muscle pathologies were all negative. Ultimately, a diagnosis of PACNS was confirmed through a stereotactic brain biopsy, which took place 3.5 years after the onset of symptoms. The patient responded favorably to treatment with glucocorticoids and cyclophosphamide.

**Conclusion:**

In summary, we have described a case of PACNS characterized by localized cerebral atrophy and tumor-like MRI findings, who was misdiagnosed as immune-associated encephalitis or mitochondrial encephalopathy for 3.5 years. We emphasize the importance of dynamic observation of MRI changes, as well as brain biopsy.

## Introduction

Primary angiitis of the central nervous system (PACNS) is a rare condition associated with high levels of disability and mortality. Its clinical presentation varies significantly depending on the size of the affected blood vessels in the brain and spinal cord, encompassing symptoms such as headaches, altered cognition, focal deficits, seizures, and visual disturbances, among others. In the United States, the estimated average incidence rate stands at 2.4 cases per 1,000,000 person-years (1). People of all ages can be susceptible to PACNS. Notably, PACNS may manifest distinctive imaging features, which can include normal findings, multiple infarctions, hemorrhages, microbleeding, demyelination, unique or multiple contrast-enhanced masses, and meningeal thickening (2). These imaging findings may resemble cerebrovascular diseases, Rasmussen encephalitis, mitochondrial encephalomyopathy, neoplasms, and more. Due to its low incidence, wide range of clinical presentations, and significant variations in neuroimaging, identifying PACNS can be challenging. Biopsy remains a critical diagnostic tool; however, many clinicians are apprehensive about its invasive nature and the possibility of not sampling affected tissue, particularly in pediatric patients.

In this context, we present the case of a 16-year-old girl with a 3.5-year history of recurring headaches and epilepsy. Due to atypical clinical manifestations and nonspecific MRI findings, she was misdiagnosed for a very long time. Final diagnosis of PACNS was confirmed through histological analysis, which revealed lymphocytic vasculitis. In summary, we underscore the importance of considering PACNS as a potential diagnosis in teenagers with epilepsy who exhibit brain lesions on magnetic resonance imaging (MRI) scans.

### Case description

**Figure 1:**
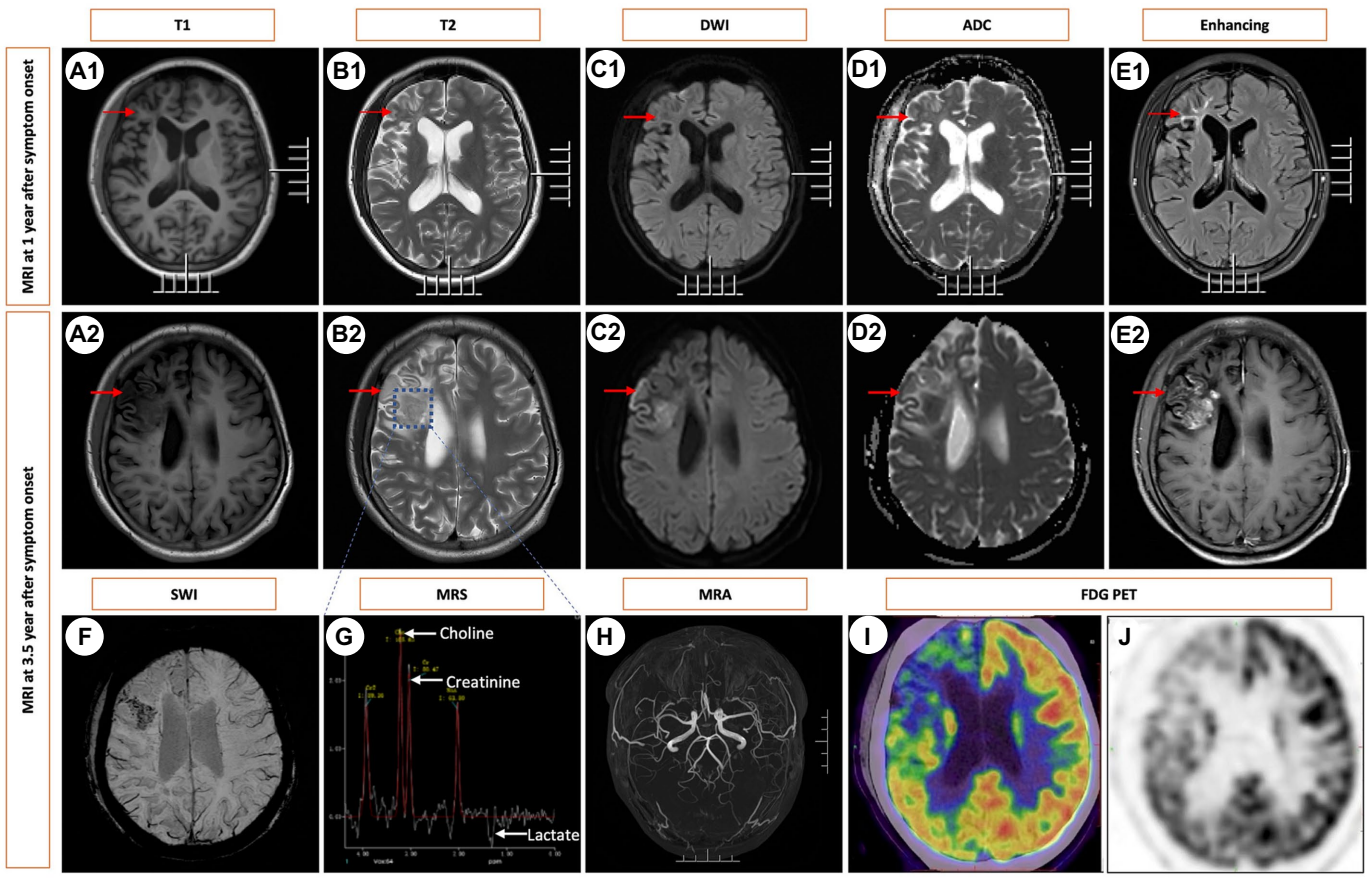
Sequential Brain MRIs Following Symptom Onset. Brain images obtained at 1 year **(A1–E1)** and 3.5 years after the onset of symptoms **(A2–E2)** and **(F–J)**. T1-weighted MRI revealed hypointense white matter lesions in the frontoparietal lobes (indicated by arrowheads) along with localized cerebral atrophy **(A1)**. This lesion exhibited hyperintensity on T2 WI **(B1)**, hypointensity on DWI **(C1)**, and hyperintensity on the ADC **(D1)**, along with enhancement on contrast-enhanced MRI **(E1)**. At the 3.5-year mark from symptom onset, a solitary mass was identified in the right frontal lobe, exhibiting hyperintensity on T2 WI and contrast enhancement following gadolinium injection, accompanied by edema but without any mass effect. The corresponding images are shown in A2-E2. SWI showed microbleeds within the mass **(F)**. MRS indicated an increased choline/creatinine ratio within the lesion **(G)**. MRA did not reveal any vascular abnormalities **(H)**. FDG-PET scans revealed atrophy in the right frontal lobe, right temporo-insula, right parietal, and right occipital cortex, along with multiple calcifications and reduced glucose metabolism, predominantly in the right frontal lobe and right temporo-insula **(I,J)**. T1, T1-weighted imaging. T2, T2-weighted imaging. DWI, diffusion-weighted imaging. ADC, apparent diffusion coefficient. SWI, susceptibility weighted imaging. MRS, magnetic resonance spectroscopy. MRA, magnetic resonance angiography. FDG PET-CT, ^18^F-fluorodeoxyglucose PET-computed tomography.

A 16-year-old girl, without any significant family medical history, presented with a three-and-a-half-year history of headaches and epilepsy. The initial manifestation was a tic in her left eyelid, which subsequently spread to her left oral region, left upper limb, and left lower limb, eventually affecting both limbs. Alongside these motor symptoms, she also experienced episodes of altered consciousness. No family history or significant medical history were reported, except her motor skills are poorer compared to her peers. Her neurological examination yielded normal results. Comprehensive laboratory tests, including blood gas analysis, routine blood counts, chemistry panel, liver function tests, kidney function assessment, and ammonia levels, all returned unremarkable results. Lactate acid in blood at rest was normal (0.91 mmol/L, normal: 0.7–2.2 mmol/L). Notably, her serum homocysteine levels were slightly elevated at 21.05 μmol/L (normal range: 5–15.0 μmol/L), though this finding had limited clinical significance. Extensive infectious evaluations, including cerebrospinal fluid analysis, HIV, and treponema pallidum testing, along with an autoimmune encephalitis panel, revealed no abnormalities. Additionally, autoimmune serologic tests, such as antinuclear, anticardiolipin, ANCA, rheumatoid factor, and antineuronal antibodies, were all negative. Her endocrine evaluations yielded normal results. Electroencephalography demonstrated asymmetric slow waves on the right side, interspersed with sharp waves, particularly in the frontotemporal region. A T2-weighted MRI obtained 1 year after the onset of symptoms displayed hyperintensity in the frontoparietal lobes, along with localized cerebral atrophy. This lesion exhibited hypointensity on diffusion-weighted imaging (DWI) and hyperintensity on the apparent diffusion coefficient (ADC) map (Figures 1A1–E1).

Immune diseases were actively sought but no evidence found. However, doctors cannot definitively exclude this possibility. A brain biopsy was considered. However, due to its invasive nature, her parents declined the brain biopsy in favor of diagnostic immunotherapy. Then, the girl underwent treatments with immunoglobulin (0.4 g/Kg per day for 5 days) and rituximab (0.6 g per 6 months) successively. Her symptoms were initially controlled, but soon relapsed. Mitochondrial encephalomyopathy was considered at another hospital due to the clinical presentation and findings on brain MRI. Consequently, a muscle biopsy was conducted. However, no specific changes indicative of mitochondrial encephalopathy, such as damaged red blood cells, were observed under a light microscope. Furthermore, both mitochondrial DNA (mtDNA) and nuclear DNA (nDNA) sequencing using biopsied muscle failed to reveal any pathogenic variants associated with mitochondrial encephalopathy, effectively ruling out this possibility.

**Figure 2:**
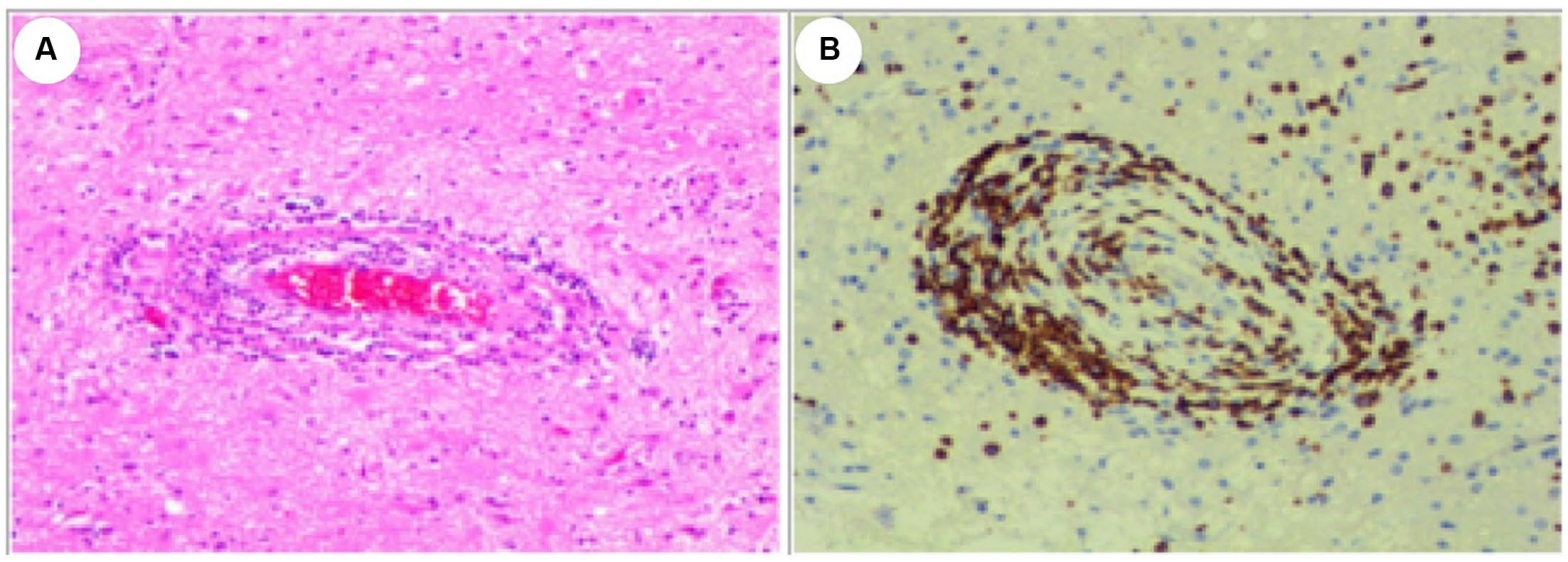
Histological Examination of the Lesion. **(A)** Hematoxylin and eosin (HE) stain. **(B)** CD3 positive. The examination revealed dense perivascular chronic inflammation characterized by the presence of lymphocytes. Lymphocytes predominantly infiltrated and disrupted the walls of small blood vessels. Original magnification×100.

**Figure 3:**
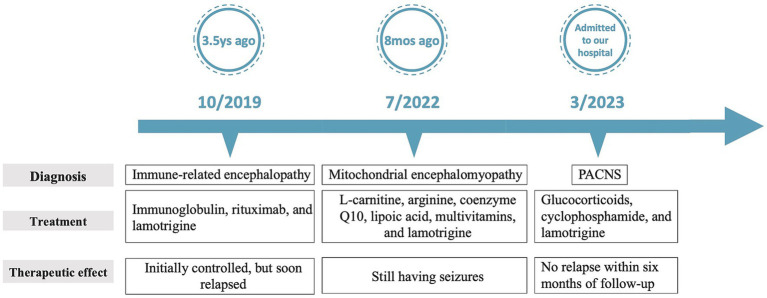
A time course of events.

Serial MRI assessments were performed every 6 months. A subsequent MRI, conducted 3.5 years after symptom onset, revealed a right frontal lesion that exhibited hypointensity on T1-weighted imaging (T1 WI), hyperintensity on T2-weighted imaging (T2 WI) and FLAIR, along with enhancement on contrast-enhanced MRI (Figures 1A2–E2). Magnetic resonance spectroscopy (MRS) indicated an increased choline/creatinine ratio within the lesion (Figure 1G). MR angiography (MRA) did not reveal any involvement of large-or medium-sized blood vessels (Figure 1H). FDG-PET scans revealed atrophy in the right frontal lobe, right temporo-insula, right parietal, and right occipital cortex, along with multiple calcifications and reduced glucose metabolism, predominantly in the right frontal lobe and right temporo-insula (Figures 1I,J). To make a definite diagnosis, stereotactic brain biopsy was performed. Histological analysis revealed lymphocytic vasculitis (Figure 2). Previous extensive diagnostic work-up has ruled out secondary causes of CNS vasculitis, such as systemic vasculitis, infections, neoplasms. So, PACNS was confirmed. Subsequently, the patient received treatment with intravenous methylprednisolone pulses, starting at 500 mg per day and reducing the dose by half every three days. This was followed by a three-month course of gradually tapered oral glucocorticoids, combined with monthly intravenous cyclophosphamide at a dose of 0.4 g. At the six-month follow-up, there was no evidence of relapse. A time course of events can be found in Figure 3.

## Discussion

PACNS is an uncommon inflammatory condition primarily affecting various sizes of blood vessels within the central nervous system (CNS), and it manifests with a wide range of clinical symptoms, including seizures, cognitive impairment, altered consciousness levels, and focal neurological deficits. The specific features of the disease can vary depending on the predominant size of the affected brain vessels. In a study conducted in 2016, Boysson et al. compared the characteristics of PACNS patients with isolated small-vessel involvement to those with large/medium-vessel involvement. They discovered that patients with isolated small-vessel PACNS tended to be younger and presented with a higher frequency of seizures, cognitive impairment, altered consciousness, and dyskinesias, although they experienced fewer strokes at the time of diagnosis (3, 4). Our current case, involving a teenager presenting with headache and epilepsy, aligns with the findings mentioned above.

MRI imaging in PACNS can display a variety of patterns, including normal findings, multiple infarctions, hemorrhages, microbleeds, areas of demyelination, solitary or multiple contrast-enhanced masses accompanied by perilesional edema and mass effect, as well as meningeal thickening, among others (2). Cortical atrophy was identified in some patients (5). But the pathogenesis of atrophic changes is poorly understood. We speculate that the cerebral atrophy may be attributed to secondary ischemic and hypoxic changes in neuronal cells and demyelination of nerve fibers, both consequent to vasculitis. Further investigation is warranted to elucidate the pathogenetic mechanisms. MRI manifestations can often resemble conditions such as cerebrovascular diseases, Rasmussen encephalitis, mitochondrial encephalomyopathy, neoplasms, and more. Therefore, it’s very easy to misdiagnose this condition in clinical practice. In the early stages of our patient’s illness, MRI showed infiltrative white matter lesions, coupled with localized cerebral atrophy. However, at the 3.5-year mark from symptom onset, the MRI indicated a solitary contrast-enhanced mass with vasogenic edema. It indicates the importance of dynamic observation of MRI changes.

A tumor-like presentation is a rare occurrence in PACNS, accounting for approximately 11.76% of cases as reported previously (4). This specific presentation of PACNS is primarily associated with involvement of small-sized blood vessels. Consequently, the clinical profile of patients with a tumor-like presentation of PACNS closely resembles that of PACNS patients with isolated small-vessel involvement. Previous research findings have indicated that patients with a tumor-like presentation of PACNS typically exhibit predominant involvement of small-sized blood vessels. This aspect often necessitates pathological confirmation because conventional vascular imaging techniques, such as MRA and computed tomography angiography (CTA), typically yield negative results (4). Digital subtraction angiography (DSA) is a helpful study in the imaging of PACNS, especially in PACNS with the medium and smaller arteries involvement. However, vasculopathy shown in DSA cannot be differentiated from noninflammatory etiologies. High resolution vessel wall MRI (VW-MRI) is emerging as a valuable tool for distinguishing between causes of intracranial arterial narrowing. In PACNS, VW-MRI typically reveals uniform, smooth enhancement and thickening of the affected arteries. Beyond differentiating PACNS from conditions like atherosclerosis or Reversible Cerebral Vasoconstriction Syndrome, VW-MRI can also guide the selection of the most affected vessels for tissue biopsy, potentially improving diagnostic accuracy (5–7). Histological studies have shown that a lymphocytic vasculitis pattern is more commonly observed in these cases. This indicates that the clinical presentation, MRI findings, and pathological features of PACNS correspond to each other and largely depend on the size of the affected blood vessels.

The diagnosis of PACNS is established by considering isolated neurological symptoms, evidence of brain vessel involvement on neurovascular imaging, or a CNS biopsy, and by ruling out all conditions that may mimic or contribute to CNS vasculitis (8). Biopsy remains the gold-standard method for achieving a definitive diagnosis, as it provides direct evidence of vascular involvement. In this case report, this adolescent presented with a combination of symptoms, including headaches, epilepsy, and focal hemispheric lesions displaying unilateral asymmetry. Initially, although lactate acid in blood at rest was normal, mitochondrial encephalomyopathy was still considered due to the clinical presentation and findings on brain MRI. However, muscle pathologies of the patient did not show specific changes indicative of mitochondrial encephalopathy, such as damaged red blood cells. In addition, both mtDNA and nDNA sequencing failed to reveal any pathogenic variants associated with mitochondrial encephalopathy, effectively ruling out this possibility. Additionally, the MRI’s tumor-like presentation and an increased choline/creatinine ratio observed in the lesion on MRS raised suspicion of neoplastic involvement. Given the challenges in distinguishing between malignant neoplasms and tumor-like PACNS before biopsy, histological confirmation became imperative. A stereotactic brain biopsy of the right frontal region was performed, revealing a dense perivascular chronic inflammation characterized by lymphocytic infiltration. This infiltration was accompanied by local brain tissue necrosis, strongly indicative of PACNS. An extensive diagnostic work-up was completed to rule out secondary causes of CNS vasculitis, such as systemic vasculitis, infections, neoplasms. Consequently, a definitive diagnosis of PACNS was established. Subsequently, the patient was treated with glucocorticoids in combination with cyclophosphamide. There was no evidence of relapse at the six-month follow-up. However, the long-term prognosis remains uncertain.

In summary, we have presented a case of unilateral hemispheric PACNS, which was conclusively diagnosed through a brain biopsy. The evolving changes in serial brain MRI scans as the disease progressed were also illustrated to underlines the importance of dynamic observation of MRI changes. When young individuals present with seizures, exhibit negative neurovascular imaging results, and display unilateral abnormalities on MRI, it is prudent to consider the possibility of isolated small-vessel PACNS. In such cases, a biopsy becomes essential to definitively exclude other potential diagnoses.

### Patient perspective

“In 2019, when I was just 13 years old, I experienced my first seizure. Before the seizure occurred, I felt dizzy and had chest congestion. It began with my left eyelid twitching, followed by twitching in my left mouth, and then it gradually spread to my left upper limb, left lower limb, and eventually affected both of my limbs. I lost consciousness during this episode, as my classmates later recounted. Approximately 5 min later, I regained consciousness. Following this incident, I underwent a thorough medical evaluation. Doctors identified a lesion in my brain, but its nature remained a mystery. Despite being prescribed lamotrigine, I continued to experience seizures, occurring approximately twice a year, much to the concern of my parents. In 2023, both my parents and I decided to follow the doctor’s recommendation to undergo a biopsy to establish a definitive diagnosis. Following the biopsy, I received treatment that yielded positive results. I’ve since returned to school and have remained seizure-free. I make regular visits to my doctor every 3 months, hoping to remain seizure-free in the future.”

## Data availability statement

The datasets presented in this article are not readily available because of ethical and privacy restrictions. Requests to access the datasets should be directed to the corresponding author.

## Ethics statement

Ethical review and approval was not required for the study on human participants in accordance with the local legislation and institutional requirements. Written informed consent from the patients/participants or patients/participants’ legal guardian/next of kin was not required to participate in this study in accordance with the national legislation and the institutional requirements. Written informed consent was obtained from the minor(s)’ legal guardian/next of kin for the publication of any potentially identifiable images or data included in this article.

## Author contributions

LQ: Writing – original draft, Writing – review & editing. MH: Writing – review & editing. WL: Writing – review & editing.
